# Mothers' intentions to vaccinate their children for COVID-19

**DOI:** 10.3934/publichealth.2023016

**Published:** 2023-03-29

**Authors:** Shruthi Venkatesh, Alexandra Gill, Lauren Kim, Stacey N Doan

**Affiliations:** 1 Department of Psychology, University of North Carolina, Greensboro; 2 Department of Psychological Science, Pomona College, Claremont, CA; 3 Department of Psychological Science, Claremont Mckenna College and Department of Population Sciences, City of Hope National Medical Center

**Keywords:** intention to vaccinate, children, mothers, COVID-19

## Abstract

Parents' intentions to vaccinate their children is an important area of investigation in light of the COVID-19 pandemic. There is a growing body of research examining factors that influence parents' vaccine intentions. The current study investigated factors that would influence maternal intent to vaccinate their children for COVID-19, shortly before the CDC approved vaccines for children 11 and younger. We had a sample of n = 176 mothers (Mchildage = 71.63 months, 52% White) from California fill out an online survey during February–April 2021. Our results suggest that perceived COVID-19 threat predicts mothers' intention to vaccinate their children (b = 0.370, p < 0.001), controlling for mothers' age, socioeconomic status, race, and child age. Child age (b = 0.027, p = 0.008), SES (b = 0.396, p = 0.018), and child previous flu shot (b = 0.725, p < 0.001) also positively predicted mothers' intention to vaccinate their children. Results are discussed in light of prior research on maternal vaccine intentions and hesitancy.

## 1. Introduction

The first cases of COVID-19, the coronavirus disease caused by SARS-CoV-2, were reported in 2019 [1]. On March 11, 2020, World Health Organization (WHO) declared COVID-19 a pandemic [2]. Researchers rapidly began COVID-19 vaccine development and clinical trials [3]. The Food and Drug Administration (FDA) set rigorous standards for vaccine developers. In December of 2020, the FDA issued the first emergency use authorization of the Pfizer-BioNTech COVID-19 vaccine in the United States for individuals aged 16 years and older, followed by the Moderna vaccine [4]. Since then, over 13 billion COVID-19 vaccine doses have been administered worldwide [5].

In October 2021, the FDA approved the COVID-19 vaccine for 5- to 11-year-old children [6]. On June 18, 2022, the Centers for Disease Control and Prevention (CDC) approved the COVID-19 vaccine for children 6 months and older, increasing eligibility to 20 million children in the United States [7]. As of August 24th, 2022, 1.2 million children aged six months to four years (7%) and 10.6 million children aged 5- to 11-year (37%) have received their initial dose of the COVID-19 vaccine; 17.5 million children aged 12- to 17-years (70%) have received their initial dose of the COVID-19 vaccine, with 15.0 million (60%) having completed the two-dose vaccination series [8].

There is a growing body of research investigating parents' intentions to vaccinate themselves and their children for COVID-19. Demographics of the family and child are seemingly the factors that influence vaccine attitudes and the likelihood of being vaccinated, such as caregiver education, race, income level, and child age. Indeed, parents and teenagers in the U.S from higher-income households were more likely to be vaccinated (73% for income above $100,000/year) than those from lower-income households (38% for income below $50,000/year) [9]. In a sample of U.S. mothers who were surveyed in February–March 2021, those with less than a graduate degree reported higher COVID-19 vaccine hesitancy (for vaccinating themselves) compared to mothers with graduate degrees [10]. Additionally, non-Hispanic Black mothers were more likely to be hesitant toward the vaccine compared to non-Hispanic White mothers [10]. Regarding vaccinating their children, caregivers across six countries demonstrated a greater willingness to vaccinate older children for COVID-19 [11]. In the United States specifically, the likelihood of child vaccination was higher for parents of older children [12]. This age-effect influencing parent vaccine attitudes is further supported by findings from a nationally representative sample of parents: four in ten parents with children under five years stated they will “definitely not” vaccinate their child for COVID-19, sharing concerns about the vaccine's newness, the limited amount of testing and research, unknown side effects, and overall safety [13]. Furthermore, previous vaccination status also influences the likelihood of children getting a COVID-19 vaccine. Children's up-to-date vaccination status and prior vaccination for the flu were positively associated with parents' willingness to vaccinate their children for COVID-19 [11],[14]. Moreover, unvaccinated parents in the U.S. surveyed in the summer of 2021 were less likely to express an intention to vaccinate their child aged 2- to 11 years old compared to vaccinated parents [15]. Thus, previous research has explored the determinants of vaccine intentions in parents of younger children that we sought to build on.

Apart from demographic variables and previous vaccination status that impact parent COVID-19 vaccine intentions, the context of the COVID-19 pandemic itself has been a major source of stress for parents and might have influenced their vaccine attitudes. This stress manifests in two forms: daily stressors (such as losing a job because of the pandemic) and perceived virus threat (fear of contracting COVID-19). Regarding daily stress, parenting during COVID-19 has greatly affected mothers in particular, as mothers traditionally take on the majority of domestic labor and childcare, which increased with the pandemic [16]. In a sample of mothers who reported quitting a job due to COVID-19, half of them did so because of the closure of schools or daycares, and one in five reported that the pandemic had a major impact on their mental health [17]. These stressors of the pandemic were positively related to anxiety and depression symptomology in a sample of U.S. mothers [18]. In addition, pandemic anxiety (fear of contracting or transmitting COVID-19) influences vaccine attitudes: mothers with low pandemic anxiety were 4.8 times more likely to report vaccine hesitancy than mothers with high pandemic anxiety [10]. Hence, while the perceived threat of COVID-19 has been measured in previous work, we sought to examine if daily COVID-19 related stress would also have an impact on mothers' intent to vaccinate their children.

Given the current cultural and socio-political climate surrounding the COVID-19 vaccine, the role of vaccinations in reducing the spread of COVID-19, as advised by medical experts throughout the pandemic, and as new COVID-19 variants emerge, it is crucial to continue to investigate factors affecting parent vaccine attitudes. These factors are broadly situated in the Health Belief Model, which purports that an individual's perceived severity, susceptibility, benefits, and barriers, spurred by a cue to action, determine their engagement in a health-promoting behavior [19]. In the context of COVID-19 pandemic (the cue to action), parents' intent to vaccinate their children (the health promoting behavior) is dependent on their perceived vaccine safety, necessity, and ideas regarding freedom of choice [14],[20]. In this study, we focus on the perceived threat of COVID-19 (i.e. feeling worried or fearful of the virus) and the perceived negative impact of COVID-19 related stressors that maps onto the perceived severity aspect of the Health Belief Model.

Compared to fathers, mothers are more likely to harbor COVID-19 vaccine hesitancy [11],[15],[21] and hence, we focused on exploring vaccine intentions in a sample of U.S mothers. Understanding mothers' perceptions of the vaccine, especially before vaccines were available for children 11-years and younger, allows for a better understanding of mothers' decisions to vaccinate their children, and the factors that could be driving the current vaccination rates. Our study hopes to add to the growing body of research on mothers' intent to vaccinate their children aged 5- to 9-years, based on previous work that has found associations with child age, previous child vaccination status, parent socioeconomic status, and pandemic anxiety. Specifically, we aimed at examining if COVID-19 related stressors would also be a predictor of maternal vaccine intentions.

## 2. Materials and methods

### 2.1. Participants and procedure

Data for this study was conducted using a cross-sectional survey method, from February–April 2021. Mothers in the study were part of a larger longitudinal study examining the effects of the COVID-19 pandemic on parenting, stress, and children's social and emotional development in mothers based in Southern California. The eligibility criterion for this overall study was that mothers were English-speaking, had children aged 4- to 9 years, and had accessibility to the Internet. They were recruited from a database of parents of about 200 parents who had participated in our studies before the COVID-19 pandemic. Data for this study was collected via Qualtrics: mothers consented to the study and completed an online survey. The procedures for this study were approved by [Institution Masked] Institutional Review Board.

### 2.2. Measures: Predictor

#### 2.2.1. Demographics

Mothers reported on their race, ethnicity (whether they identify as Hispanic or Latino/a), child age, sex, and education and income levels.

#### 2.2.2. COVID-related stressors

This was a 30-item scale to capture the impact of COVID-related stressors such as losing income, trouble concentrating on work, missing events with family, and becoming sick with COVID-19, used in prior work [18],[22], for example, “What was the impact of this stressful life event? You have spent less time interacting with your friends.” The impact of each event, if it had occurred for the participant, was rated on a scale of one (“not really negative”), two (“sort of negative” to three (“very negative”). Impact scores were summed to give a composite score for the participant, with high scores indicating more negative COVID-related stressors.

#### 2.2.3. Perceived COVID-19 threat

The Perceived Coronavirus Threat scale, developed by social psychologists at the start of the COVID-19 pandemic [23], consists of 6 items that assess participant perceptions of the extent to which they believe the coronavirus is a threat. Sample items include “I am afraid of the coronavirus”, ranging from one (“not true of me at all”) to seven (“very true of me”). A mean score was computed, with higher scores indicating higher perceived coronavirus threat. The Cronbach's alpha in our sample was 0.99.

#### 2.2.4. Vaccine attitude

Previous work that has investigated caregivers' intent to vaccinate their child for COVID-19 have found positive associations with children's previous vaccination status, specifically, if they had been vaccinated for influenza in the past 12–24 months [11],[12]. To account for prior vaccine attitudes, we asked mothers if their child had received a flu vaccine in the past six months with response options ranging from one to four: 1) I'm not sure if my child got the flu shot 2) No, my child has not gotten a flu shot and will not get one 3) No, my child had not gotten a flu shot but will get one before January 1, 2021, 4) Yes, my child got a flu shot. Here we'd like to note that “before January 1, 2021” was just before the study began and indicated the last flu season. Higher scores indicate the child had previously received the flu shot or will get one in the near future.

### 2.3. Measures: Outcome

We measured mothers' intent to vaccinate their child by asking about what they would do when there is a COVID-19 vaccine, which was modeled off of the questions used in [24], with response options being 1) I will never have my child take the COVID-19 vaccine, 2) Get my child vaccinated after it has been available for a year 3) Get my child vaccinated 6–12 months after it becomes available, 4) Get my child vaccinated 3–6 months after it becomes available, 5) Get my child vaccinated 1–2 months after it becomes available, 6) Get my child vaccinated the moment it becomes available. Here, higher scores indicate mothers' immediate willingness to vaccinate their child.

### 2.4. Statistical analysis

Variables were checked for normal distribution before analysis. In our sample, income, and education were positively correlated with each other, r = 0.62, p < 0.01 and hence were standardized and averaged to create a socioeconomic status (SES) variable. We also created a binary variable for mothers' race, with zero for White and one for non-White (all other races, such as Asian, African-American, and more than one race) mothers. We then ran correlation analysis to examine the relationship between our variables of interest, including our demographic variables, survey variables (perceived COVID-19 threat, child previous vaccination status, and COVID-related stressors), and the outcome variable (intent to vaccinate). This analysis was conducted to check for covariates before running our multiple linear regression model.

## 3. Results

Our sample included 176 mothers, Mage = 36.33 years, SDage = 5.05 years who participated in our study. They had children in the target age of 5- to 9 years, with Mage = 71.64 months, SDage = 11.19 months. Ninety-two mothers (52%) identified as Caucasian or White, and 57 (32%) identified as Hispanic/ or Latin o/a. In this sample, 88 mothers (50%) reported annual income above $80,000, and 81 had graduate degrees (46%, Master's or Doctorate degrees, see Table 1 for demographics).

Pearson's correlations revealed that mother's age, child age, SES, and race were significantly correlated with our outcome variable, the intent to vaccinate their child. Specifically, mother's age r = 0.425, p < 0.01, child's age r = 0.343, p < 0.01, and SES r = 0.523, p < 0.01 were positively associated with mother's intent to vaccinate their child. These associations of older mothers, mothers with older children, and mothers from higher SES backgrounds being more willing to vaccinate their child for COVID-19 have been shown in previous work [12],[15]. Additionally, maternal race was negatively related to the intent to vaccinate r = −0.332, p < 0.01, highlighting that non-White mothers had lower intentions to vaccinate their child. Given these associations, mother's age, child age, mother race and SES were treated as covariates and controlled for in further analysis (see Table 2 for correlations).

In addition to demographic variables, our data shows that in our sample, 76 children (43%) had received their flu shot in the previous flu season. Mother's average perceived COVID-19 threat was M = 4.84, SD = 1.40 on a scale of 1–7, and the average impact of COVID-19 related stressors was M = 22.13, SD = 10.76 with Range = 0–66.34 mothers (19%) indicated an intention of immediately vaccinating their child when the COVID-19 vaccine became available, and 26 mothers (15%) indicated their child will never get the COVID-19 vaccine.

We then ran a multiple linear regression using the LAVAAN package in R. Full Information Maximum Likelihood method (comparative results were found with list-wise deletion) was used to address missing data. Specifically, we ran a multiple linear regression using COVID-related stressors, perceived COVID threat, and vaccine attitudes to predict mothers' intent to vaccinate their child against coronavirus, controlling for maternal age, child age, SES, and mothers' race. Our main effects model revealed that child age, b = 0.027, SE = 0.010, p = 0.008; SES, b = 0.396, SE = 0.167, p = 0.018, previous vaccine attitudes, b = 0.725, SE = 0.134, p < 0.001, and perceived coronavirus threat, b = 0.370, SE = 0.086, p < 0.001 positively predicted mothers' intent to vaccinate their child for the coronavirus (see Table 3 for regression model).

**Table 1.** Participant Demographics.

Measure	*n*	% (rounded to nearest integer)
Maternal education		6
High school	10	
Some college	33	19
Community college	12	7
Bachelor's degree	40	23
Graduate degree	81	46
Maternal race		
White/Caucasian	92	52
Asian/Asian-American	17	10
Black/African-American	2	1
Multiracial/other	65	37
**Income (from 2019)**<$20,00	15	9
$20,000–$40,000	26	15
$41,000–$60,000	20	11
$61,000–$80,000	27	15
$81,000–$100,000	18	10
$101,000–$120,000	18	10
$121,000–$150,000	15	9
$151,000–$175,000	17	10
$175,000	20	11

**Table 2.** Correlation Matrix for Key Measures.

Measure	2	3	4	5	6	7	8
1. Mother age	0.228**	−0.271**	0.577**	0.241**	−0.049	0.291**	0.425**
2. Child age		−0.191*	0.221**	0.110	0.039	0.194*	0.343**
3. Mother race			−0.456**	−0.010	0.041	−0.272**	−0.332**
4. SES				0.234**	−0.113	0.373**	0.523**
5. Perceived COVID threat					0.254**	0.316**	0.456**
6. COVID Stressors						0.168*	0.046
7. Vaccine attitude							0.592**
8. Intent to vaccinate child							

*Note: Race was coded as 0 for White and 1 for Non-White mothers. *p < 0.05 **p < 0.01 ***p < 0.001. Fit for model R2 = 0.54, CFI and TLI = 1.00.

**Table 3.** Predicting mothers' intent to vaccinate their child for COVID-19.

		CI_95%_			
Predictor	*b*	Lower	Upper	SE	*z*	*p*
Mother Age	0.027	−0.027	0.080	0.027	0.097	0.332
Child Age	0.027	0.007	0.047	0.010	2.633	0.008**
Mother Race	−0.329	−0.816	0.158	0.248	−1.325	0.185
SES	0.396	0.069	0.723	0.167	2.37	0.018*
Perceived COVID-19 Threat	0.370	0.201	0.538	0.086	4.298	0.000***
COVID Stressors	−0.010	-0.031	0.011	0.011	−0.974	0.33
Vaccine attitude	0.725	0.463	0.988	0.134	5.418	0.000***

*Note: Race was coded as 0 for White and 1 for Non-White mothers. *p < 0.05 **p < 0.01 ***p < 0.001. Fit for model R2 = 0.54, CFI and TLI = 1.00.

## 4. Discussion

In this study, we examined predictors of maternal intent to vaccinate their 5- to 9-year-old children for COVID-19 before vaccines were available to children 11 years and younger. This study contributes to and expands on the growing literature in this domain. Child age, child previous vaccination status, and maternal socioeconomic status positively predicted mothers' willingness to vaccinate their child for COVID-19, suggests that mothers of older children and mothers from higher socioeconomic backgrounds were more likely to be willing to vaccinate their children for COVID-19 when the vaccine becomes available. Moreover, if the child had already gotten a flu shot, then that increased the likelihood of mothers reporting being willing to vaccinate their child for COVID-19. These findings are akin to those of previous studies [9]–[12],[14],[15], which have also found that mothers have higher intentions of vaccinating their children for COVID-19 if they are older, come from higher SES backgrounds, and have previously received a vaccine.

As extant research suggests, parents perceive the COVID-19 vaccines as relatively new with unknown side effects, which are factors that contribute to their vaccine hesitancy [11],[14],[15]. Drawing from a lens of perceived severity using the Health Belief model, in our sample, mothers who perceived COVID-19 as a threat were more willing to vaccinate their children. Our study extends the results from prior work on maternal pandemic anxiety and vaccine hesitancy for oneself [10] to include maternal perceived pandemic threat to vaccine hesitancy for her child. We did not find an effect of COVID-related stressors on maternal vaccine intentions, highlighting the significance of perceiving the virus as a threat in predicting vaccine intentions over that of the contextual stressors of the pandemic.

While this study has corroborated similar findings for previous research in this area, we note that one of first studies to examine parental perceptions of the COVID-19 vaccine was only published in 2021 [14] and the average child age was 12. A literature review reveals only a handful of studies looking at specifically parental perceptions of vaccinations for their children. Moreover, in past research, most of the sample was White (over 91% of the Ruggiero et al. (2021) study identified as White). Our study is unique in that our sample of children is particularly young, and our sample is ethnically diverse. Finally, we also believe that given the study was done before vaccines were widely available for children, it provides an early look at parental perceptions, which may change over time.

## 5. Limitations

Notwithstanding the strengths, we acknowledge that our study has a comparatively small sample size and represents mothers' from highly educated and higher income backgrounds (with about 50% having graduate degrees and incomes above $80,000 a year), which could restrict the generalizability of the findings. Additionally, our survey is cross-sectional and only measured maternal vaccine intentions in early 2021. Now that vaccines are widely available for children 11 and younger, future research should examine actual vaccination rates in children with mothers who had expressed varying vaccine intentions from diverse socioeconomic backgrounds to further qualify and nuance our understanding of this critical phenomenon. Furthermore, we included only the perceived severity aspect of the Health Belief Model in testing maternal vaccine intentions. Additional work is required to examine other elements of this model (such as perceived barriers, benefits or susceptibility) in evaluating mothers' willingness to vaccinate their children for COVID-19.

## 6. Conclusions

Our study highlights important factors that would influence mothers' intention to vaccinate their children in a period before COVID-19 vaccines were authorized for children younger than 11 years. Specifically, it extends the findings of previous research on vaccine intentions in mothers, by illuminating that child age, child previous vaccination status for the flu, and maternal perceived COVID-19 threat as vital predictors of their intent to vaccinate their preschool and school-age children. Despite the pandemic being a significant stressor for mothers, in our sample, stress did not affect their vaccine intentions; instead, higher COVID-19 threat, was more predictive of vaccination intentions. In light of the misinformation circulated during the pandemic [25], it is crucial to investigate the factors that influence vaccine acceptance in mothers, especially as they tend to be the decision drivers for getting children vaccinated.
